# Synthesis and Structural Identification of a Biaryl Ether-Linked Zearalenone Dimer

**DOI:** 10.3390/molecules23102624

**Published:** 2018-10-12

**Authors:** Julia Keller, Luisa Hantschke, Hajo Haase, Matthias Koch

**Affiliations:** 1 Department Analytical Chemistry, Reference Materials, Bundesanstalt für Materialforschung und -prüfung (BAM), Richard-Willstätter-Straße 11, 12489 Berlin, Germany; juliakeller19@yahoo.de (J.K.); luisa.hantschke@web.de (L.H.); 2 Department of Food Chemistry and Toxicology, Technische Universität Berlin, Gustav-Meyer-Allee 25, 13355 Berlin, Germany; haase@tu-berlin.de

**Keywords:** mycotoxin, dimerization, HRMS, NMR

## Abstract

A new dimer of the food-relevant mycotoxin zearalenone was isolated after electrochemical and chemical oxidation. The structure was determined as a 16-*O*-15′-biaryl ether-linked dimer based on spectroscopic analyses (^1^H- and ^13^C-NMR, COSY, HMBC, and HSQCAD) and high-resolution mass spectrometry analysis (Q-TOF).

## 1. Introduction

The fungal secondary metabolite zearalenone (ZEN) is found worldwide and is primarily produced by *Fusarium* species [1,2,3]. Often found in common crops like corn, wheat, rice, soybeans, sorghum, spices, or walnuts, it poses a health risk to human and animals [4,5,6]. As mycoestrogen, it causes swelling of the uterus and vulva, infertility, and atrophy of ovaries reported in swine and cattle [7,8]. Several metabolites derived from plants, fungi, and mammalian metabolism are part of ongoing research due to unknown toxic effects and occurrence [9,10,11,12].

Oxidative reactions of ZEN lead to hydroxylated species obtained from in vitro assays with liver microsomes of rodent and non-rodent liver cells [13,14]. A recent study proposed the production of numerous hydroxylated as well as new dimeric species of ZEN by using electrochemistry coupled to mass spectrometry [15]. The production, isolation, and structural elucidation of the predominant dimeric species is now achieved.

## 2. Results and Discussion

**Figure 1:**
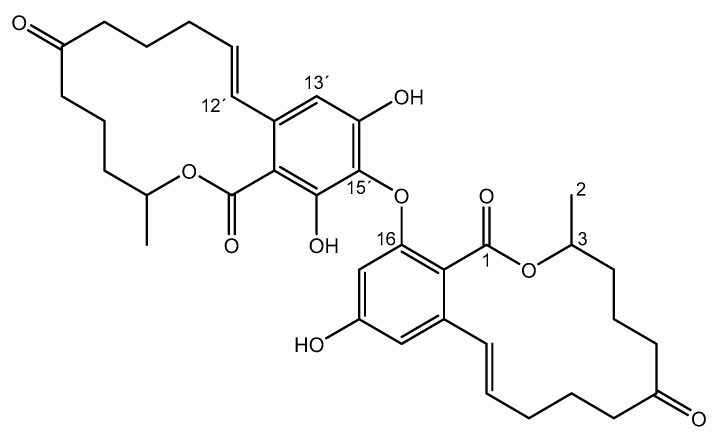
Structure of compound **1.**

Compound **1** was obtained as a pale orange solid substance after electrochemical and chemical oxidation of ZEN with a molecular formula of C_36_H_42_O_10_, which was measured by high-resolution mass spectrometry with *m/z* 633.2658 [M − H]^−^ in an ESI negative ionization mode (theoretical exact mass *m/z* 633.2702), as previously described [15]. The MS/MS measurements of compound 1 revealed a fragment with *m/z* 589.2756, which is due to the loss of CO_2_, and a fragment with *m/z* 565.2746 because of a loss of C_3_O_2_. The signal with *m/z* 491.1658, corresponds to a loss of C_8_H_4_O_2,_ which led to the fragment *m/z* 447.1763 after the loss of CO_2_ (Supplement Figure S1). The assumed fragments and their chemical formulas with theoretical exact masses are shown in Figure 1.

The ^1^H-NMR spectrum of compound 1 measured at 400 MHz in MeOH-*d*_4_ gave the following information with 1.00–2.85 (m, H, *J* = 6.8 Hz), 1.34 (d, 3H, *J* = 6.2 Hz), 1.39 (d, 3H, *J* = 6.4 Hz), 5.06–5.16 (m, 1H), 5.20–5.36 (m, 1H), 5.71 (ddd, 1H, *J* = 4.0, 9.8, 15.6 Hz), 5.77 (d, 1H, *J* = 2.1 Hz), 6.03 (d, 1H, *J* = 4.4, 9.6, 14.6 Hz), 6.34–6.41 (m, 2H), 6.42 (s, 1H), and 6.58 (d, 1H, *J* = 2.1 Hz). The ^13^C-NMR measurements with 100 MHz revealed the following chemical shifts with 20.2, 21.0, 22.1, 22.6, 23.4, 32.4, 35.9, 37.6, 38.3, 43.9, 44.7, 73.7, 74.3, 100.8, 104.3, 106.7, 126.4, 130.2, 134.4, 137.2, 139.4, 158.0, 161.0, 172.1, 213.8, and 214.1 ppm (Supplement Figures S2 and S3). The assignments of the carbons and protons are summarized in Table 1.

The ^1^H-NMR and COSY spectra revealed two aromatic systems (Supplement Figures S2 and S4). One of these aromatic systems contained two protons (d 6.58 ppm, 1H, *J* = 2.1 Hz, and d 5.77 ppm, 1H, *J* = 2.1 Hz) while the second aromatic system had only one proton (s 6.42 ppm, 1H). From this observation, the structures connected over the C-C linkages 15, 15′, 13, 13′, and 13, 15′ can be excluded since these dimers would have two aromatic systems with only one proton. Thus, only dimers having an C-O-C ether bridge between the monomers are possible. The COSY spectrum showed nearly identical chemical structures for the spectral part of the molecule (Supplement Figure S4). As a result, the dimerization of two ZEN molecules is only likely over an ether-link between 14-O-13′, 14-O-15′, 16-O-13′, or 16-O-15′. The HMBC spectrum (8 Hz) indicates a common coupling partner of the single aromatic proton and 12′-H, which is located in the aromatic-olefinic region (Supplement Figure S5). Due to the spatial proximity, it should be at the singlet (6.42 ppm) and, as a result, act around the 13′-H position. Consequently, an O-15′-linkage is conceivable.

**Figure 2:**
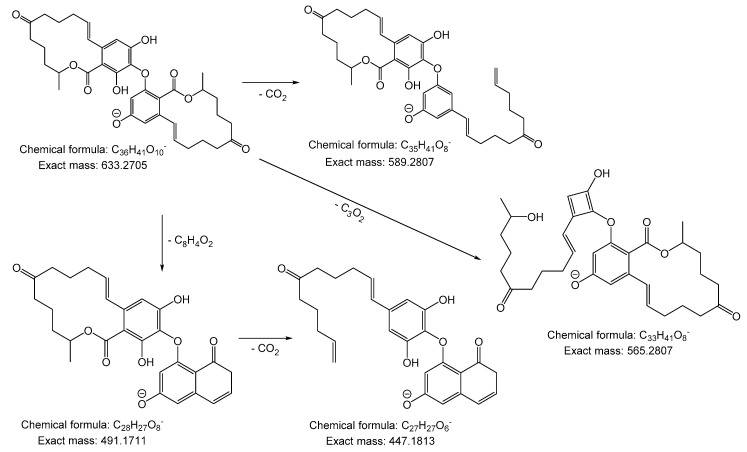
Postulated fragments of the ZEN-dimer, according to the MS/MS measurements with a molecular structure and formula along with the theoretical exact masses.

The observed significant difference of the chemical shifts of 3-H and 3′-H indicated a different chemical environment. For a C-14 link, the closer chemical environment of 3-H and 3′-H would be very similar. A C-16 link, on the other hand, would be a greater steric influence and inductive effects would occur. Thus, the compound 16-O-15′-biaryl ether bond is the most likely structure, which is shown in Figure 2. Whether this dimer can be found naturally in food or feed remains to be analyzed in detail. Especially in plants, lichen, bacteria, and fungi regio-selective and stereo-selective biaryl C-C and biaryl ether C-O-C linkages are often found and it is conceivable that dimers of ZEN might not be uncommon in nature [16].

## 3. Materials and Methods

### 3.1. Chemicals and General Experimental Procedures

Zearalenone with a purity over 98% was obtained from Fermentek (Jerusalem, Israel) and Cerium(IV)sulfate was purchased from Sigma-Aldrich (Steinheim, Germany). Ultrapure water was generated by a Seralpur PRO 90 CN system (Ransbach-Baumbach, Germany). All standard chemicals were of p.a. grade and all solvents were of an HPLC grade. Electrochemical oxidation was achieved by using the Roxy^®^ system synthesis cell (Antec, Zoeterwoude, The Netherlands) equipped with a platinum working electrode. The HPLC system used for fractionation consisted of an Agilent 1200 series autosampler, a 1260 series pump, a 1200 series diode array detector, and a column oven. A Macherey-Nagel Nucleosil C18 100-5 150 × 4.6 mm column (Düren, Germany) was used. The TripleTOF^®^ 6600 Quadrupole Time-Of-Flight (QTOF) mass analyzer (Sciex, Darmstadt, Germany) was operated in negative ionization mode and 10 µM of the dimer sample were dissolved in methanol with 0.1% of formic acid. The used parameters were as follows:

Gas temperature 350 °C, ion source gas 1 (nitrogen) 20 L/min, ion source gas 2 (nitrogen) 15 L/min, curtain gas (nitrogen) 20 L/min, and ion spray voltage floating −4500 V. The MS/MS-spectrum was recorded in the targeted MS/MS mode with the following parameters: De-clustering potential (−15 V), collision energy (−40 V), and TOF Masses 100–640 Da. Confirmation of the 16-O-15′-biaryl ether-linked dimer structure was conducted by nuclear magnetic resonance spectroscopy (NMR). The NMR spectra were recorded in methanol-*d*_4_ on an Agilent 400-MR NMR spectrometer (Agilent Technologies, Waldbronn, Germany) at 30 °C. For the measurements, the ATB 5mm-probe head was operated at 399.8 MHz for ^1^H and 100.5 MHz for ^13^C data.

### 3.2. Electrochemical and Chemical Production of ZEN Dimer

*Electrochemical:* The optimal potential was tested by taking aliquots after different time points and using potentials of 0, 1.0, 1.4, and 1.8 V vs. Pd/H_2_. For the electrochemical oxidation, 80 mL of 250 µM ZEN in acetonitrile/water (50/50, *v*/*v*) was stirred for 48 h using 1.4 V vs. Pd/H_2_. The solution was subsequently evaporated to dryness by using a rotary evaporator and dissolved in water/acetonitrile (65/35, *v*/*v*) for HPLC fractionation.

*Chemical:* For the oxidative production of ZEN dimers, Ce(IV)sulfate was used. About 100 mg of ZEN and 350 mg of Ce(IV)sulfate were dissolved in 200 mL of acetonitrile/water (50/50, *v*/*v*) and stirred for two hours at 70 °C. Subsequently, the sample was stirred for 24 h at room temperature and a white precipitate was formed. The yellow solvent mixture was extracted three times with 20 mL of ethyl acetate. After the extraction, the ethyl acetate was colored yellow and the acetonitrile water mixture was colorless. After evaporation, a deep orange and highly viscous fluid was obtained. After freeze-drying, a pale orange solid was formed with a yield of 10%.

### 3.3. Purification of ZEN Dimer

For the separation of ZEN dimers, an already described HPLC method was adapted [15] by using a flowrate of 1.2 mL/min and an isocratic eluent consisting of water/acetonitrile, 65/35, *v*/*v* without modifiers. The ZEN dimer was isolated by collecting the fraction between 15.4 min and 16.2 min of the retention time by using a Foxy^®^ R1 fraction collector (Teledyne ISCO, Nebraska, NE, USA). The purity of the dimer was determined to be 92% based on DAD spectra by using a wavelength at *λ* = 254 nm (Supplement Figure S7).

## 4. Conclusions

A new dimeric species of the food-relevant mycotoxin zearalenone was synthesized electrochemically and chemically with Ce(IV)sulfate and structurally identified. Among other possible dimers, the occurrence of the 16-*O*-15′-biaryl ether-linked dimer in food and feed is conceivable because the dimerization of phenolic compounds is often observed in plants, fungi, or lichen.

## Figures and Tables

**Table 1:** ^1^H-NMR and ^13^C-NMR data of **1** (400/100 MHz, Methanol-*d*_4_).

Position	^1^H (ppm)	^13^C (ppm)
1, 1′	-	213.8, 214.1
2	1.39 (d, *J* = 6.4 Hz, 3H)	35.9
3	5.20–5.36 (m, 1H)	73.7
10, 10′	-	32.4, 32.4
11	6.03 (ddd, *J* = 4.4, 9.6, 14.6 Hz, 1H)	134.4
12	6.38–6.41 (m, 1H)	130.2
13	6.58 (d, *J* = 2.1 Hz, 1H)	106.7
14	-	161.0
15	5.77 (d, *J* = 2.1 Hz, 1H)	100.8
2′	1.34 (d, *J* = 6.2 Hz, 3H)	35.9
3′	5.06–5.16 (m, 1H)	74.3
11′	5.71 (ddd, *J* = 4.0, 9.8, 15.6 Hz, 1H)	137.2
12′	6.34–6.37 (m, 1H)	126.4
13′	6.42 (s, 1H)	104.3

## Supplementary Materials

The following are available online at http://www.mdpi.com/1420-3049/23/10/2624/s1, Figure S1. MS/MS fragmentation spectrum of the ZEN dimer obtained with Q-TOF in negative ionization mode. Figure S2. ^1^H-NMR spectrum of the 16-*O*-15′-biaryl ether-linked zearalenone dimer. Figure S3. ^13^C-NMR spectrum of the 16-*O*-15′-biaryl ether-linked zearalenone dimer. Figure S4. COSY spectrum of the 16-*O*-15′-biaryl ether-linked zearalenone dimer. Figure S5. HMBC spectrum of the 16-*O*-15′-biaryl ether-linked zearalenone dimer. Figure S6. HSQC spectrum of the 16-*O*-15′-biaryl ether-linked zearalenone dimer. Figure S7. HPLC-DAD chromatograms (λ = 254 nm) of the zearalenone dimer reaction mixture before fractionation (top) and after fractionation (bottom).
