# Supplementary material for: Synthesis and Structural Identification of a Biaryl Ether-Linked Zearalenone Dimer
Source: Molecules. 2018 Oct 12;23(10):2624. doi: 10.3390/molecules23102624 (PMC6222810; doi:10.3390/molecules23102624)
Supplement: Supplementary file 1 [file molecules-23-02624-s001.pdf]

Supplementary Materials of **“Synthesis and Structural Identification of a Biaryl Ether-Linked Zearalenone Dimer”** by Julia Keller, Luisa Hantschke, Hajo Haase and Matthias Koch.

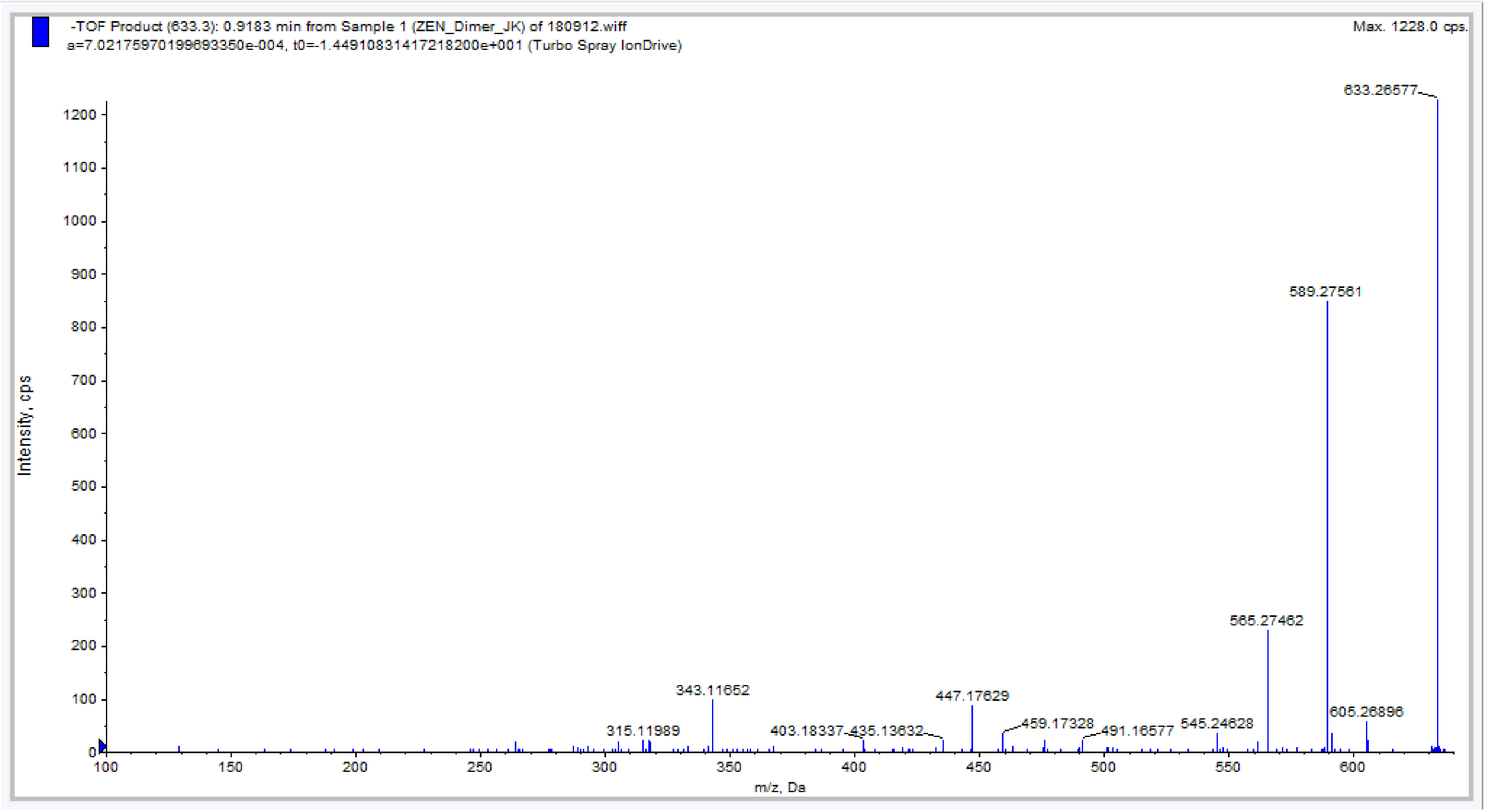

Fig. S1 MS/MS fragmentation spectrum of the ZEN-dimer obtained with Q-TOF in negative ionization mode.

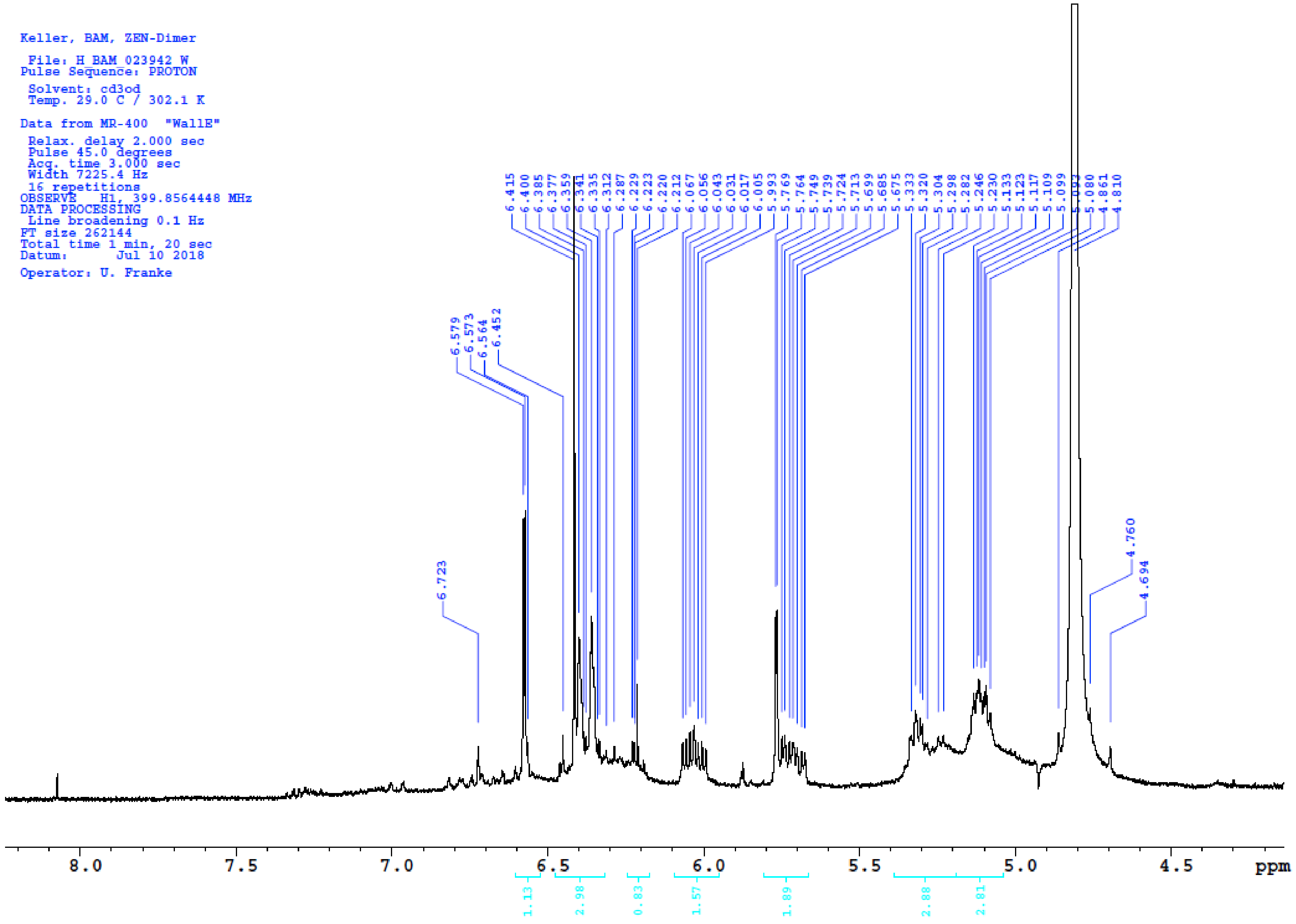

Fig. S2 ^{1}H-NMR spectrum of the 16-O-15′-biaryl ether-linked zearalenone dimer

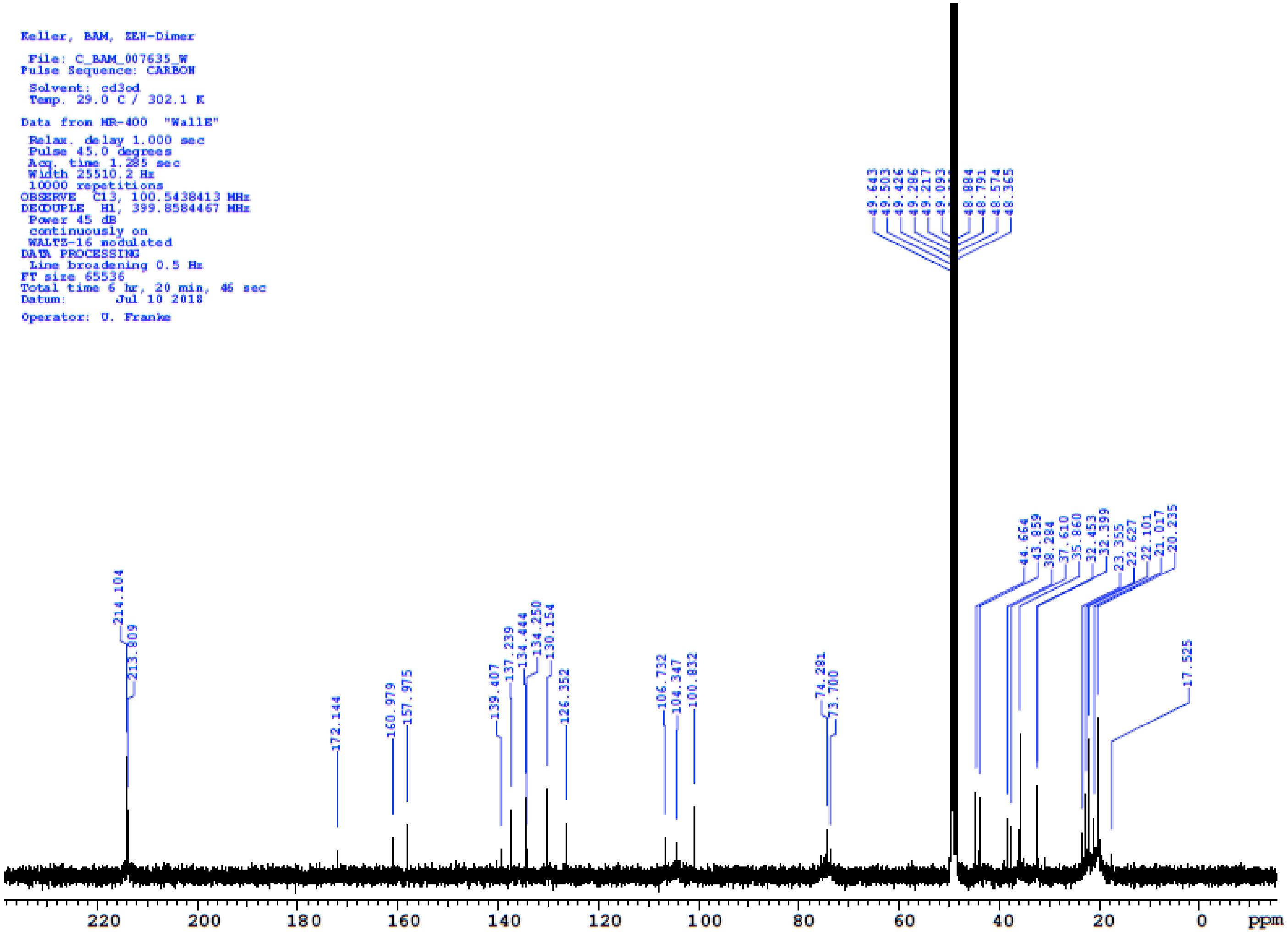

Fig. S3 ^{13}C-NMR spectrum of the 16-O-15′-biaryl ether-linked zearalenone dimer

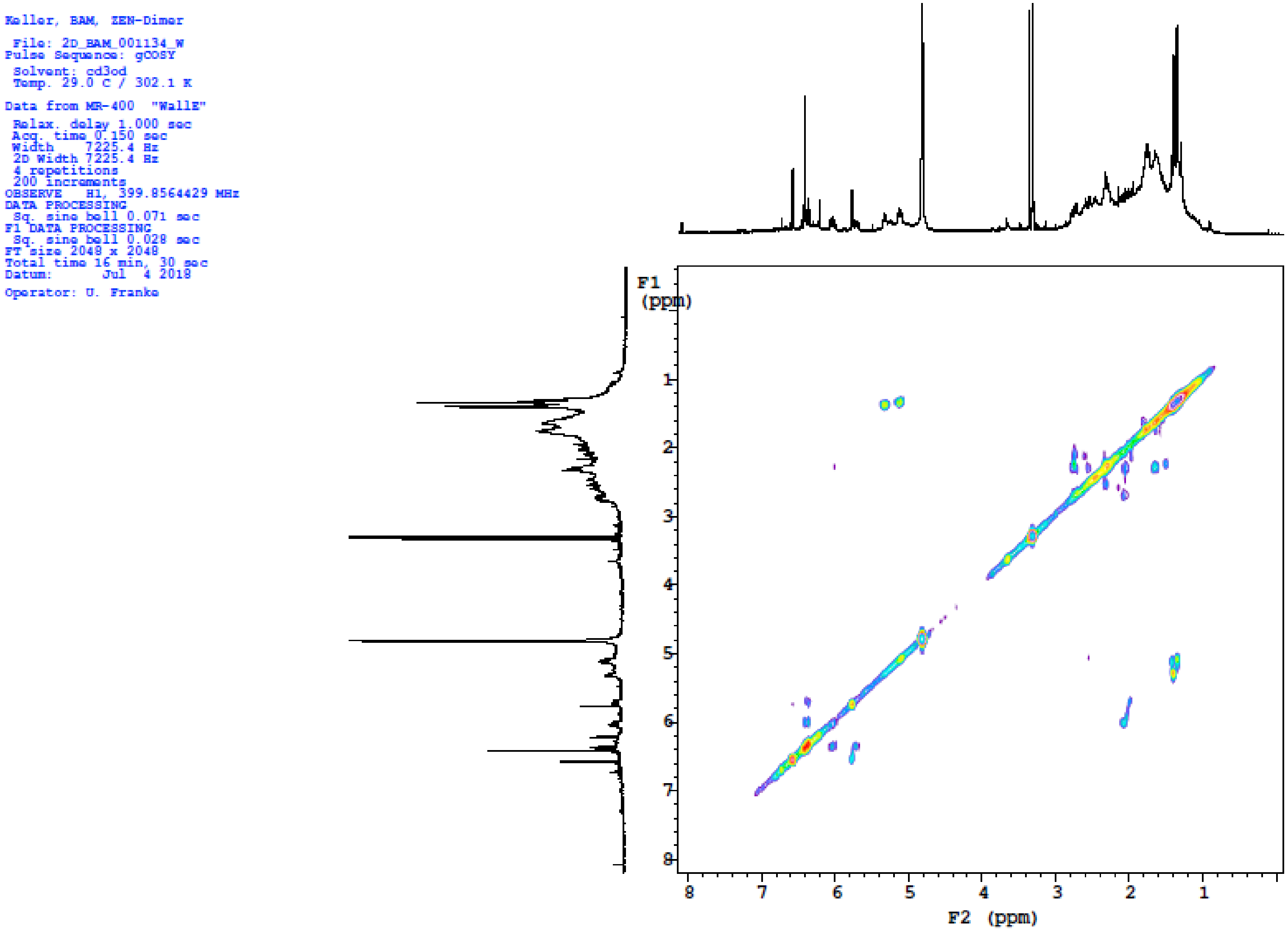

Fig. S4 COSY spectrum of the 16-O-15′-biaryl ether-linked zearalenone dimer

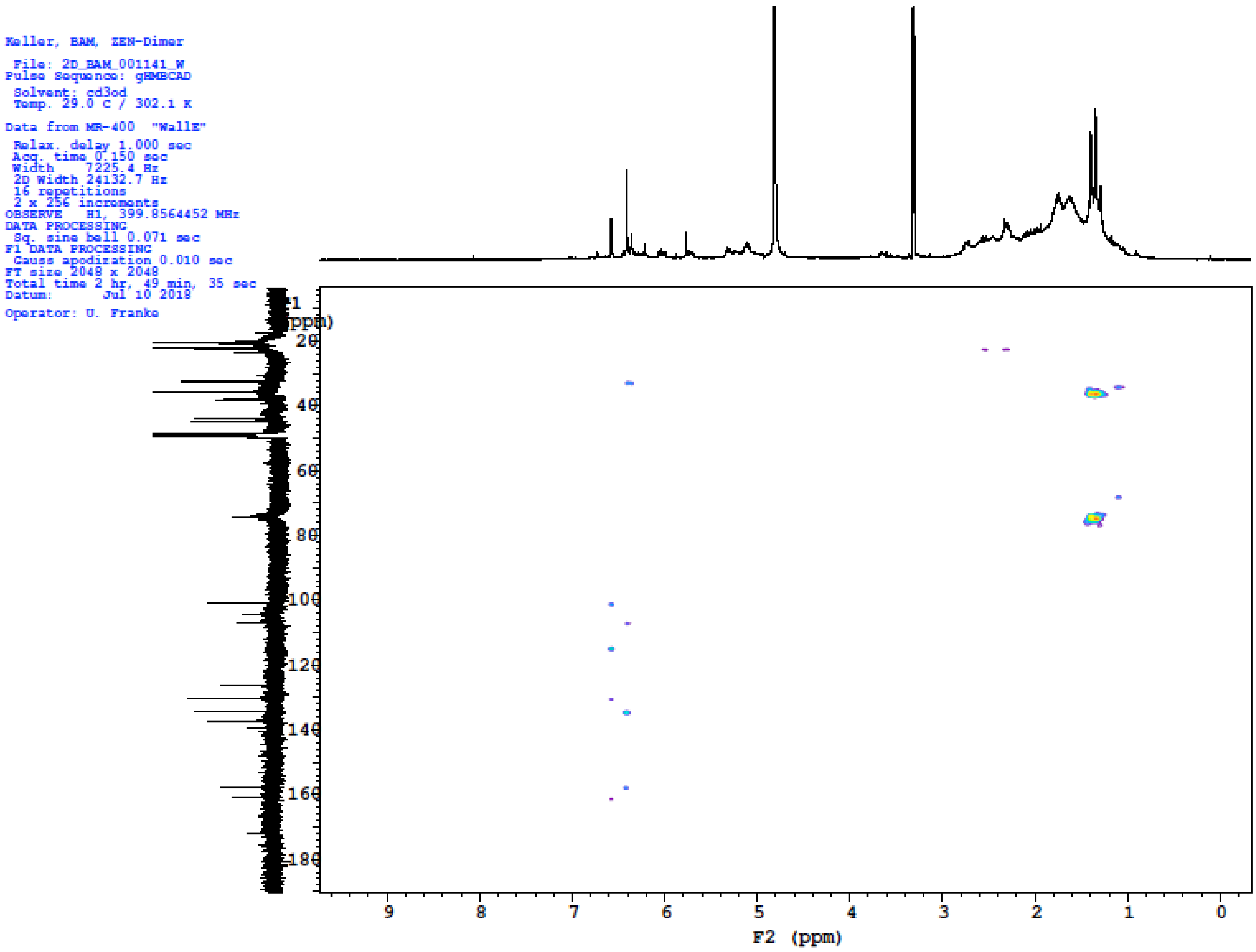

Fig. S5 HMBC spectrum of the 16-O-15´-biaryl ether-linked zearalenone dimer

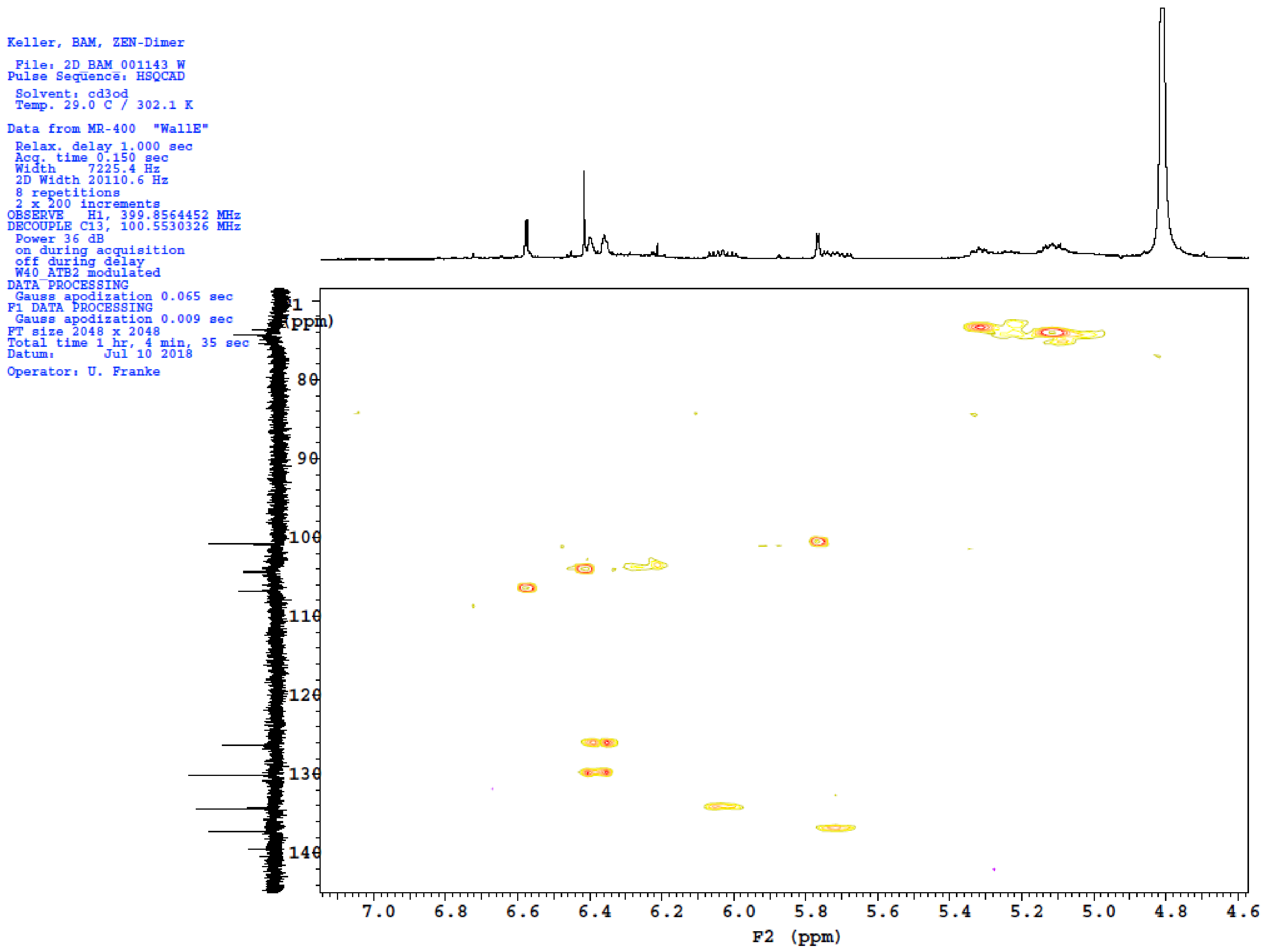

Fig. S6 HSQC spectrum of the 16-O-15′-biaryl ether-linked zearalenone dimer

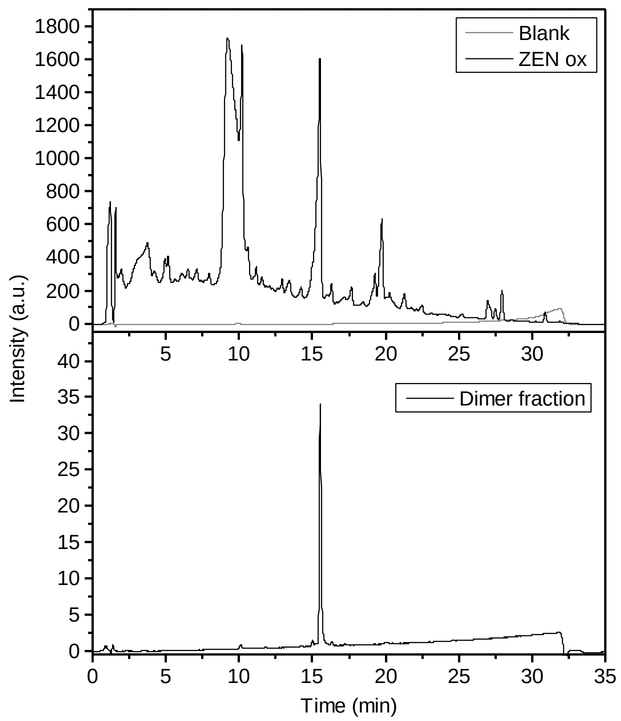

Fig. S7 HPLC-DAD chromatograms (λ=254 nm) of the zearalenone dimer reaction mixture before fractionation (top) and after fractionation (bottom)
